# The characteristics of screening and confirmatory test results for HIV in Xi’an, China

**DOI:** 10.1371/journal.pone.0180071

**Published:** 2017-07-07

**Authors:** Linchuan Wang, Kai-Hua Zhou, He-Ping Zhao, Ji-Han Wang, Hai-Chao Zheng, Yan Yu, Wei Chen

**Affiliations:** 1 The First Affiliated Hospital of Xi’an Jiaotong University, Xi’an, Shaanxi Province, China; 2 Hospital of Xi’an Jiaotong University,Xi’an, Shaanxi Province, China; 3 Honghui Hospital, Xi’an JiaotongUniversity, Xi’an, Shaanxi Province, China; 4 Xi'an Center for Disease Control and Prevention,Xi’an, Shaanxi Province, China; central virology lab, ISRAEL

## Abstract

**Objectives:**

Individuals with recent or acute HIV infection are more infectious than those with established infection. Our objective was to analyze the characteristics of detection among HIV infections in Xi'an.

**Methods:**

A 4^th^-generation kit (Architect HIV Ag/Ab Combo) and three 3^rd^-generationEIA kits (WanTai, XinChuang and Livzon) were used for HIV screening. Overall, 665 individuals were identified as positive and were tested by western blotting (WB). The characteristics of the screening and confirmatory tests were analyzed, including the band patterns, the early detection performance and the false-positive rates.

**Results:**

In total, 561 of the 665 patients were confirmed as having HIV-1 infection, and no HIV-2 specific band was observed. Among these 561 WB-positive cases, reactivity to greater than or equal to 9 antigens was the most commonly observed pattern (83.18%), and the absence of reactivity to p17, p31 and gp41 was detected in 6.44%, 5.9% and 2.86% of the cases, respectively. Two cases were positive by the 4^th^-generation assay but negative by the 3^rd-^generation assay for HIV screening and had seroconversion. The false-positive rate of the Architect HIV Ag/Ab Combo (22.01%) was significantly higher than those of WanTai (9.88%), XinChuang (10.87%) and Livzon (8.93%), p<0.05.

**Conclusion:**

HIV infection in Xi'an is mainly caused by HIV-1, and individuals are rarely identified at the early phase. Although the false-positive rate of the 4^th^-generation assay was higher than that of the 3^rd^-generation assay, it is still recommended for use as the initial HIV screening test for high-risk individuals. In Xi’an, a 3rd-generation assay for screening could be considered.

## Introduction

Since the first case of AIDS was identified in 1981 in the USA [1], HIV has spread at an alarming rate around the world, with approximately 36.9 million people infected worldwide [2]. Previous studies [3–5] have shown that individuals with recently or acutely acquired HIV are more infectious than those with established infection. Therefore, early screening tests for individuals in a high-risk setting are key to managing HIV infection. Tests for the screening and diagnosis of HIV infection have developed to the 5^th^ generation over the past 3 decades [6, 7].

Currently, the 3^rd^-and 4^th^-generation tests are the preferred assays for HIV screening and diagnosis. The 3^rd^-generation assay can detect HIV-1/2 IgG/IgM antibodies within a window of approximately 3–4 weeks after exposure. The 4^th^-generation assay combines HIV-1/2 antibodies and p24 antigen detection with a window of approximately 2–3 weeks [6–9]. Although the p24 antigen is associated with acute HIV infection [9] and the 4^th^-generation assay has better sensitivity during early infection than the 3^rd^-generation assay [6–13], nonspecific reactions are often detected using the 4^th^-generation assay [14–18]. In the study, 665 patients were confirmed by WB at the Xi'an Center for Disease Control and Prevention (CDC), and we analyzed the characteristics of the screening test, the confirmatory test and the patients’ follow-up results.

## Materials and methods

### Study population

The study populations were from three hospitals in Xi’an, including the First Affiliated Hospital of Xi’an Jiaotong University, the Second Affiliated Hospital of Xi’an Jiaotong University and Shaanxi Province People’s Hospital. In accordance with the Chinese National guidelines for the detection of HIV/AIDS (2009), a total of 665 patients with positive HIV initial screening and retesting results in Xi'an in 2015 were included in the WB test at the Xi'an CDC. The median age was 36 years old (range: 7–86), and the male/female ratio was 565/100. Among the subjects, 309, 162, 138 and 56 patients were initially screened by the Architect HIV Ag/Ab Combo, WanTai, XinChuang and Livzon, respectively. The data from the initial screening, the duplicate retesting and the WB tests in the study were from the Xi'an CDC.

### Third-generation assay

The three 3^rd^-generation kits, i.e., WanTai (WanTai Biological Pharmacy Enterprise Co, Ltd, Beijing, China), XinChuang (InTec Products, INC, XiaMen, FuJian, China) and Livzon (LivzonDiagnostics Inc., Zhuhai, China), were based on a double-Ag sandwich enzyme immunoassay to detect HIV-1/2 IgG and IgM antibodies. The HIV antigens of the kits are mixtures of HIV-1 gp120, HIV-1 gp41 and HIV-2 gp36. All tests were performed and interpreted in accordance with the manufacturer’s recommendations. An S/CO ≥1 and an S/CO<1 were defined as a positive result and a negative result, respectively.

### Fourth-generation assay

The Architect HIV Ag/Ab Combo (Abbott Diagnostics, Abbott Park, IL) relies on a chemiluminescent immunoassay (CMIA) and was designed for the simultaneous detection of HIV-1 gp41 and HIV-2 gp36 antibodies and HIV-1 p24 antigen. The test was performed and interpreted in accordance with the manufacturer’s recommendations. A cut-off index (COI) ≥1 and a COI <1 were defined as a positive result and a negative result, respectively.

### Western blot

Western blot HIV1/2 BLOT 2.2 (MP Biomedicals, Singapore) is a confirmatory test for the HIV-1/2 antibody, with separated HIV-1 gene product groups of *gag* (p17, p24, p39, p55), *pol* (p31, p51, p66), *env* (gp120, gp160, gp41) and HIV-2-specific antigen immobilized on the membrane. The test was performed in accordance with the procedures described in the instructions. The results were interpreted according to the Chinese CDC criteria: a positive result required the presence of at least two bands, including two *env* bands (HIV-1: gp41 and gp120/gp160 and HIV-2: gp36 and gp105/gp140) or one *env* band and one p24 band. An indeterminate result was defined as the presence of a band profile that did not meet the positive criteria, and a negative result was the absence of any of the specific bands.

### Follow-up protocols

For indeterminate or negative WB results, three WB tests were required at months 1, 3 and 6 of follow-up by the Chinese CDC. In our hospital, the follow-up protocols were as follows. (1) If the individual was not considered high-risk for HIV infection and the screening value ranged from 1 to 3 (3^rd^-generation EIA) or 1 to 5 (4^th^-generation assay), then three WB tests were performed at months 1, 3, and 6 of follow-up. (2) For an individual in a high-risk setting or with screening results ≥3 (3^rd^-generation EIA) or ≥5 (4^th^-generation assay), the WB test was performed at week 2, week 4, month 3 and month 6 of follow-up.

### Statistical analysis

Statistical analyses were performed using SPSS 13.0 (serial number 5026743; SPSS Inc., Chicago, IL, USA), and the WB test was used as the gold standard in the study. Chi-square tests were used to compare the rates, and a p-value < 0.05 was considered statistically significant.

### Ethics statement

The study was deemed exempt from review by the Ethics Committee of the First Affiliated Hospital of Xi'an Jiaotong University because of its retrospective nature.

## Results

### The comparison of the false-positive rate and the positive predictive value (PPV)

**Fig 1:**
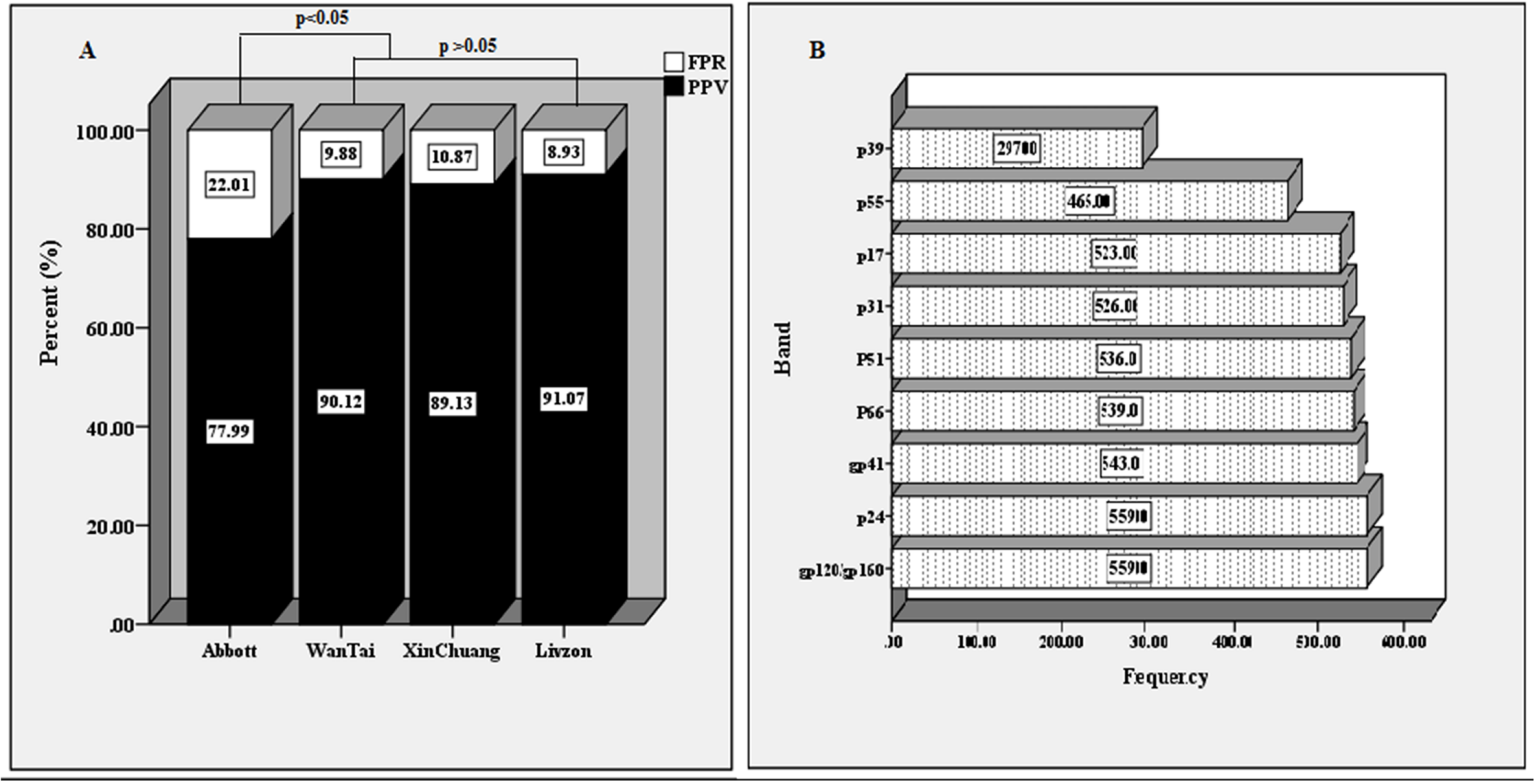
The comparison of four kits performance and the frequency of different band detection. A, The comparison of false-positive rate and positive predictive value (PPV) between a 4^th^ generation assay(Architect HIV Ag/Ab Combo) and three 3^rd^generation EIAs (WanTai, XinChuang and Livzon).Note: PPV was positive predictive value, FPR was false-positive rate.B, The frequency of different band was detected in the patients with initial WB positive results.

Overall, 559 of the 665 patients were confirmed as HIV antibody-positive by the initial WB test. All 79 patients with a WB-negative result and 25 of the 27 patients with a WB-indeterminate result were confirmed as negative by at least three WB tests. Two of the 27 patients with an indeterminate WB result (a gp120/gp160 band) had seroconversion. Overall, the PPVs of the Architect HIV Ag/Ab Combo, WanTai, XinChuang and Livzon were 77.99%, 90.12%, 89.13%, and 91.07%, respectively. Comparing the false-positive rates of the four kits, no significant differences were observed among the three 3^rd^-generation kits (p >0.05), but the false-positive rate of the Architect HIV Ag/Ab Combo (22.01%) was significantly higher than those of WanTai (9.88%), XinChuang (10.87%) and Livzon (8.93%), p<0.05 (Fig 1A and Table 1).

**Table 1:** The results of Western blot and follow-up.

screening positive n = 665	Western blot		
	Positive, n(%)	Negative, n(%)	Indeterminate, n(%)
Abbott (n = 309)	240 (77.67%)	54 (17.48%)	15 (4.85%)*
WanTai (n = 162)	145 (89.51%)	9 (5.56%)	8 (4.94%)*
XinChuang (n = 138)	123 (89.13%)	13 (9.42%)	2 (1.45%)
Livzon (n = 56)	51 (91.07%)	3 (5.36%)	2 (3.57%)

### The characteristics of the western blot band profiles

A total of 559 patients were confirmed as HIV-1 antibody-positive by the initial WB test, and the HIV-2-specific band was not observed in the study. Among the results, 11 band patterns were detected. Reactivity to all of the bands was the most commonly observed pattern (53.13%), reactivity to 9 antigens (absence of reactivity to p39) was observed in 30.05% of the cases, and the absence of reactivity to p17, p31 and gp41 was noted in 6.44%, 5.9% and 2.86% of the cases, respectively. The characteristics of the band profiles showed that the majority of the patients in the study had established HIV infections. The detection rates for the gp160/gp120, gp41, p66, p51, p31, p24, p17, p39 and p55 bands were 100% (559/559), 97.14% (543/559), 96.42% (539/559), 95.89% (536/559), 94.1% (526/559), 100% (559/559), 93.56% (523/559), 53.13% (297/559) and 83.18% (465/559), respectively (Table 2, Fig 1B). A total of 27 patients with a sole band were identified as indeterminate in the initial WB test. The p24 band was detected in 21 patients, and the gp120/gp160 band was detected in 6 cases.

**Table 2:** Frequency of 11 band patterns detected in 559 patients with initial WB positive results.

WB band profile	Frequency (n = 559)				
	Abbott	WanTai	XinChuang		Livzon
p24 p17 p55 p39 p31 p51 p66 gp41 gp120/gp160	125	80		60	32
p24 p17 p55 p31 p51 p66 gp41 gp120/gp160	67	50		39	12
p24 p17 p31 p51 p66 gp41 gp120/gp160	29	10		13	3
p24 p31 p51 p66 gp41 gp120/gp160	4	0		1	0
p24 p17 p51 p66 gp41 gp120/gp160	1	0		0	2
p24 p51 p66 gp41 gp120/gp160	2	2		2	0
p24 p31 p51 gp41 gp120/gp160	0	0		0	1
p24 p66 gp41 gp120/gp160	3	1		1	0
p24 p51 gp41 gp120/gp160	0	1		0	0
p24 gp41 gp120/gp160	1	1		0	0
p24 gp120/gp160	8	0		7	1

### Patients with a WB-negative result or with a WB-indeterminate result

In the study, 79 and 27 patients, respectively, were identified as negative and indeterminate by the first WB. Among these subjects, 87 patients did not seroconvert according to three WB tests (months 1, 3 and 6), 17 of the 19 patients in high-risk settings were confirmed as negative through four WB tests (week 2, week 4, month 3 and month 6), and 2 of the 19 patients in high-risk settings showed seroconversion by WB retesting at week 2.

Case 1: the first screening was negative by XinChuang (0.61), but 2 days later, the retesting on another sample was positive using WanTai (3.06), Livzon (5.79) and the Architect HIV Ag/Ab Combo (64.33) but negative by XinChuang (0.8). The gp120/gp160 band in the first WB test and the p24, p66, gp41, and gp120/gp160 bands in the second WB test one week later were observed. Case 2: the first sample tested positive by the Architect HIV Ag/Ab Combo (12.57) but retested as negative using XinChuang (0.17). One week later, another sample was positive in both XinChuang (12.17) and the Architect HIV Ag/Ab Combo (127.81). The gp120/gp160 band for the first WB and p24, p31, p51, gp41, and gp120/gp160 bands for the second test one week later were observed.

### The consistency and PPV of the four kits at different screening values

When the Architect HIV Ag/Ab Combo screening positive cases were retested using a 3^rd^-generation kit, at COI values of 1–5, 5–30 and ≥ 30, the consistencies were 6.82%, 64.1% and 100%, respectively. When the 3^rd^-generation kits were used for both screening and retesting, at screening S/CO ratios of 1–3, 3–10 and ≥10 for WanTai, the consistencies were 23.08%, 40% and 100%, respectively. For XinChuang, they were 20%, 46.88% and 100%, and for Livzon,they were 20%, 37.5% and 100%, respectively (Table 3).

**Table 3:** The consistency and PPV of four kits at different screening values.

Screening positive			Retesting positive	Consistency %	Western blot confirmed		
Used kit	Value	Number	Number		FP(n)	TP(n)	PPV(%)
Architect HIV Ag/Ab Combo							
	1.00–5.00	44	3	6.82	44	0	0
	5.00–30.00	39	25	64.1	24	15	38.46
	≥30.00	226	226	100	0	226	100
WanTai							
	1.00–3.00	10	3	23.08	10	0	0
	3.00–10.00	12	8	40	6	6	50
	≥10.00	140	140	100	0	140	100
XinChuang							
	1.00–3.00	8	2	20	8	0	0
	3.00–10.00	17	15	46.88	7	10	58.82
	≥10.00	113	113	100	0	113	100
Livzon							
	1.00–3.00	4	1	20	4	0	0
	3.00–10.00	20	12	37.5	1	19	95
	≥10.00	32	32	100	0	32	100

Note: FP was false positive; TP was true positive.

**Fig 2:**
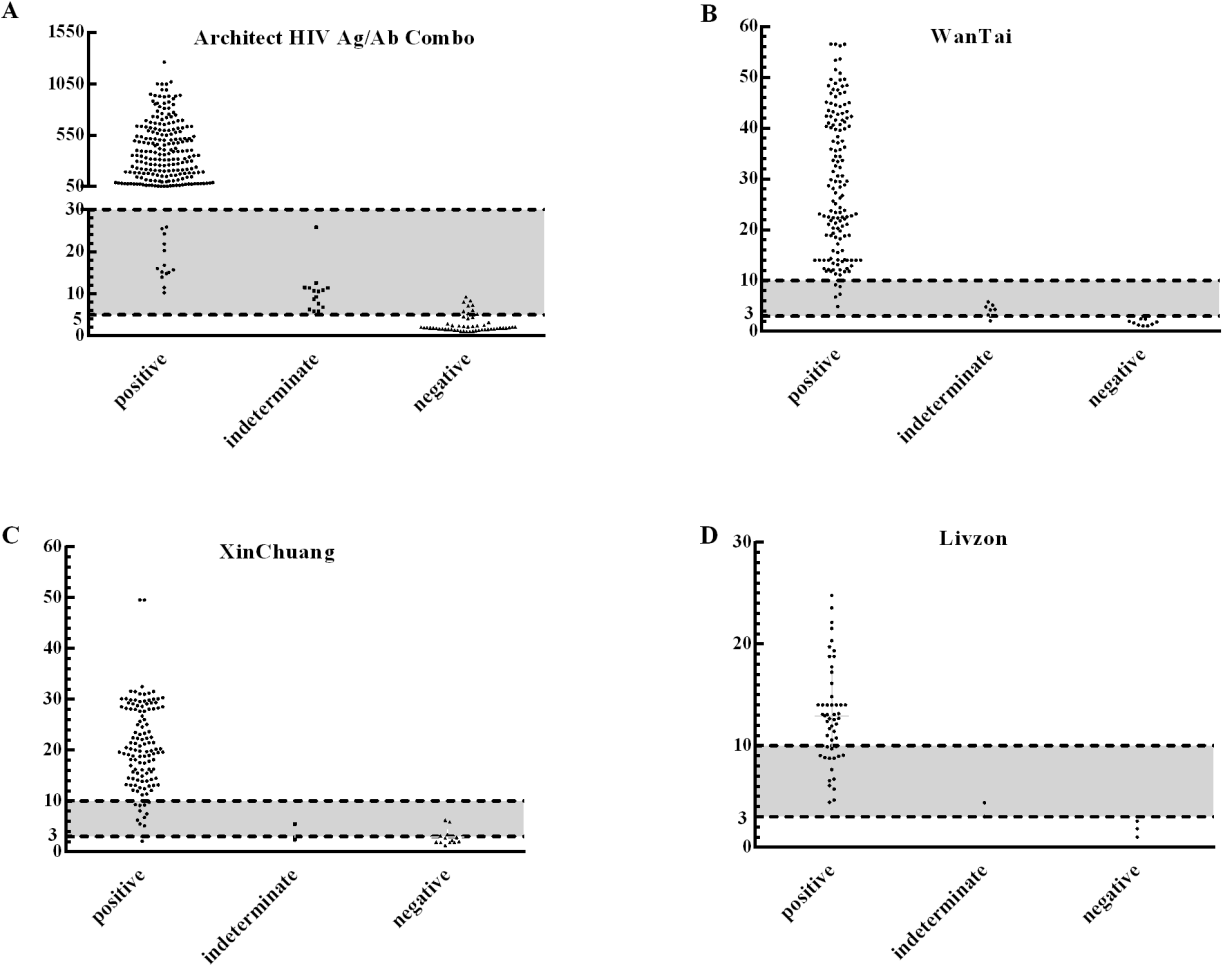
Distribution of screening values using different kits in the first WB negative, indeterminate and positive result.

In the first WB,at COI <5 or S/CO < 3 and at COI ≥30 or S/CO ≥10, all of the patients were found negative and positive, respectively (Fig 2).When the screening values for the Architect HIV Ag/Ab Combo were1-5, 5–30 and ≥30, the corresponding PPVs were 0, 38.64%, and 100%. At S/CO ratios of 1–3 and ≥10, the PPVs of the 3^rd^-generation kits were 0 and 100%, respectively. At S/CO ratios of 3–10, the PPVs of WanTai, XinChuang and Livzon were 50%, 58.82% and 95%. In the study, 63.46% (66/104) of the false-positive results were distributed at COI 1–5 or S/CO 1–3 (Table 3).

## Discussion

To determine whether an individual is infected with HIV, an initial screening test, duplicate retests and confirmatory tests should be performed. People are often concerned about the specificity of HIV screening kits. Currently, antigens such as gp41 and gp36 (Abbott Architect HIV Ag/Ab Combo and Roche Elecsys HIV Combi), gp120, gp41 and gp36 (domestic kit and Bayer ADVIA centaurg HIV 1/O/2 enhanced), gp160, gp41 and gp36 (Genscreen™ ULTRA and VIDAS DUO Ultra) are usually used to detect HIV-1/2 antibodies [13, 19, 20] with excellent sensitivity [8, 10–13, 19, 21, 22]. Many factors, such as influenza vaccination [14,15], rheumatoid factors, autoimmune diseases [15], parasitic infection [16], and pregnancy [18], may lead to poor specificity in HIV screening by the 4^th^-generation assay and an indeterminate result for the WB test [23, 24].

Jensen et al [25] reported that the false-positive rate of the Architect HIV Ag/Ab Combo was 31.39% (1163/3705). In our studyit was 22.01% (68/309) but was significantly higher than the 3^rd^-generation kit, which was consistent with previous reports [21, 26]. We found that higher screening values were correlated with an increased consistency and PPV. In the study, 63.46% of the false-positive results were distributed at COI 1–5 or S/CO 1–3. When the screening values were at COI values ≥30 or S/CO ratios ≥10, the consistency and PPV were both 100%. However, at COI <5 or S/CO < 3, the PPV was 0, and the consistency was only 6.82%~23.08%.

Previous studies [27, 28, 29] have shown that the variations in the WB band pattern were associated with the stages of HIV infection. Antibodies to *gag* antigens were detectable earlier than were antibodies to *env* antigens [27, 28].The absence of reactivity to *gag* antigen p17, *pol* antigen p31[27] or *env* antigen gp41 [30] were often observed among seroconverters. WB profiles with the presence of p17 may constitute a predictor of established HIV infection [27]. In the study, reactivity to more than or equal to 9 antigens was the most commonly observed WB pattern, occurring in 83.18% of cases, and the absence of reactivity to p17, p31 and gp41 antigens was noted in only 6.44%, 5.9% and 2.86% of cases. According to the criteria of Fiebig et al [29], 2, 33 and 526 of the 561 HIV infection cases in this study can be classified as stage IV, V and VI, respectively, implying that the HIV infections in Xi'an were rarely detected at the early phase. The absence of reactivity to p39 or p55 antigens may be associated with disease progression [27]. In our study, p39 and p55 bands were not observed in 46.87% and 16.82% of the HIV infections. Published results reported that gp160 and gp120 may be used as earlier antigens to detect the HIV antibody [28, 31–33]. In Saah et al study [28], the gpl20/160band wasdetected more early than gp41 band in HIV infectionsat the early phase,and 20 of the 23 gp41-negative cases were identifiedas havinganti-envelope antibodies (gpl20/160). In our study, the detection rates of gp41 and gp160 /gp120 bands in the HIV infections were 97.14% and 100%.

In the study, two cases with reactivity to gp120/gp160 had seroconversion at follow-up week 2, and they tested positive by the 4^th^-generation assay but negative by the 3^rd^-generation assay. This also confirmed that the addition of p24 antigen detection to the 4^th^-generation assay results in an earlier detection of HIV-1 infection compared to the 3^rd^-generation assay [34]. Currently, in China, if the WB test is indeterminate or negative for individuals with positive screening results, then three WB tests are required. Although most of these individuals are not infected with HIV, seroconversion [27, 28] may occur. Therefore, the current follow-up protocol in China is not suitable for all individuals. In particular, in individuals with a gp120/gp160 band or recent HIV exposure, frequent WB retesting during the first follow-up month, at weeks 2 and 4 for example, is needed.

China is a country that is characterized by income inequality and a low HIV prevalence. Both the 3^rd^- and 4^th^-generation assays are commonly used for HIV screening. The appropriate selection of the first HIV test is important. In non-high-risk settings, inexpensive 3^rd^-generation EIA assays with reduced false-positive results are advantageous. For the high-risk population, the 4^th^-generation assay is recommended as the initial HIV screening method to improve the detection rate at the early phase of HIV infection. In individuals who are positive for the gp120/gp160 WB band (suspicious for recent HIV exposure), two WB tests at the first month of follow-up are needed.

## Supporting information

S1 File The original data of the initial screening, retesting and WB testsin the study. The data that processed anonymously were from the Xi'an Center for Disease Control and Prevention (CDC).
(XLSX)
